# Characterizing Biochar as Alternative Sorbent for Oil Spill Remediation

**DOI:** 10.1038/srep43912

**Published:** 2017-03-08

**Authors:** Ludovica Silvani, Blanka Vrchotova, Petr Kastanek, Katerina Demnerova, Ida Pettiti, Marco Petrangeli Papini

**Affiliations:** 1 Università degli Studi di Roma “La Sapienza” Department of Chemistry, Rome, 00100, Italy; 2 University of Chemistry and Technology, Department of Biochemistry and Microbiology, Praha, Czech Republic; 3 Ecofuel, Ecofuel Laboratories, Praha, Czech Republic

## Abstract

Biochar (BC) was characterized as a new carbonaceous material for the adsorption of toluene from water. The tested BC was produced from pine wood gasification, and its sorption ability was compared with that of more common carbonaceous materials such as activated carbon (AC). Both materials were characterized in terms of textural features and sorption abilities by kinetic and equilibrium tests. AC and BC showed high toluene removal from water. Kinetic tests demonstrated that BC is characterized by faster toluene removal than AC is. Textural features demonstrated that the porosity of AC is double that of BC. Nevertheless, equilibrium tests demonstrated that the sorption ability of BC is comparable with that of AC, so the materials’ porosity is not the only parameter that drives toluene adsorption. The specific adsorption ability (mg sorbed m^−2^ of surface) of the BC is higher than that of AC: toluene is more highly sorbed onto the biochar surface. Biochar is furthermore obtained from biomaterial thermally treated for making energy; this also makes the use of BC economically and environmentally convenient compared with AC, which, as a manufactured material, must be obtained in selected conditions for this type of application.

Oil spill accidents have long-lasting effects on the ecosystem because of the dangerous substances that are released into the environment^1^. The production and consumption of petroleum products are increasing worldwide, and as a side effect of transportation, exploration and related processes, oil spills are unfortunately increasing accordingly^2^. Major oil spills, such as the 2010 Deep Water Horizon spill, have drawn public and media attention. This has highlighted the necessity to realize fast and easy handling responses with effective remediation strategies for mitigating and minimizing the negative consequences^3^,^4^.

This work is carried out in the frame of Kill Spill, a European (EU)-funded project aimed to develop highly efficient, economically and environmentally viable (bio)technological solutions for the cleanup of oil spills.

Conventional strategies for oil spill cleanup involve the use of sorbent materials; adsorption is extensively used for remediation purposes^5^,^6^ to remove a large range of contaminants such as metals^7^,^8^,^9^ and organic compounds^6^,^10^,^11^. Sorbent materials can be used in booms to remove the floating organic fraction or as an amendment to remediate the high-molecular-weight contamination in the sediment. Several sorbents are able to remove the separate phase, but they are often inadequate to sorb the dissolved contamination at low concentrations. In this regard, carbonaceous materials are the most common sorbents used for this type of application. Adsorption onto carbonaceous materials such as activated carbon (AC) has been indeed largely investigated for removing a large range of contaminants from wastewater^7^,^10^,^12^ and sediments^13^,^14^,^15^. The high sorption efficiency is caused mostly by their large surface area^16^,^17^, and their availability^18^ ensures that these carbonaceous materials are widely used for this purpose. In this regard, Biochar (BC) has been tested as new potential carbonaceous sorbent for removing oil spill contaminants. BC is generally obtained by pyrolysis of plant- and animal-based biomass^9^,^12^,^19^,^20^,^21^. BC is used as a soil amendment owing to its several positive effects such as contaminant bioavailability reduction^22^,^23^,^24^, increasing soil cation exchange capacity (CEC)^25^,^26^, mainly due to its nutrients and water holding capacity^27^,^28^,^29^.

BC is characterized by extensive surface area and high porosity; furthermore, its structured carbon matrix is similar to that of activated carbon^30^,^31^. Due to AC similarity, BC has been recently tested as sorbent material for metal removal from wastewater^9^,^30^,^32^. Furthermore, it has been demonstrated that BC is potentially able to immobilize a large range of organic and inorganic contaminants in soil^33^.

One of the most important advantages of BC is its low cost when it is obtained from energy producing processes. In this case, BC is the residue of a carbonization process, so any reuse of this material can valorize a potential waste.

Over recent years, there has been substantial state support for the production of green renewable energy from biomass in several EU countries, including Italy and Austria. This resulted in the commissioning of several industrial wood gasifiers. The gasification units generally convert wood biomass into wood gas, a syngas consisting of atmospheric nitrogen, carbon monoxide, hydrogen, traces of methane, and other gases, which, after cooling and filtering, can then be used to power an internal combustion engine or for other purposes.

Several design modifications of wood gasification technologies were developed during the 20^th^ and 21^st^ centuries; however, only a few have found their way into practice. Currently, such technologies are considered mainly as power plants designed for the production of electricity at small, decentralized sites. One example of such a plant is a wood gasification plant in Gussing, Austria where a steam-blown fluidized bed gasifier is used to turn 1760 kg of wood chips per hour into 2000 kW of electricity and 4500 kW of district heat. Another example is small ECO 180 HV modules for gasification of wood pellets, produced by the German company Burkhardt GmbH, that are being operated at several locations throughout Europe. The main advantages of such wood gas generators over petroleum fuels is their renewable character, closed carbon cycle, lower contribution to global warming, cleaner burning and possibility for integration into combined heat and power systems. However, some wood gasification systems also face problems with tar formation, mainly regarding water quality and the parameters of processed biomass.

The aim of this work was to characterize a BC obtained as residue from an ECO 180 HV wood pellet gasification module in terms of textural features and to investigate its sorption performance by measuring toluene removal from an aqueous liquid phase. BC sorption performance was investigated by kinetic and equilibrium (isotherm) tests, in water and synthetic seawater, to assess the material performances in conditions more similar to real oil spill conditions (e.g., sorbent materials in the boom). Moreover, BC sorption performance was compared with the more consolidated AC.

In this study, toluene was chosen as a target contaminant because it is soluble in aqueous phase. Toluene is a moderately mobile and soluble hydrophobic organic contaminant (HOC); nevertheless, by knowing its behavior, it is possible to generate useful information on the more hydrophobic oil components’ behavior, including long-chain alkanes and polycyclic aromatic hydrocarbons (PAHs).

## Materials and Methods

### Materials

#### Activated Carbon

Norit activated carbon, type Darco (Sigma Aldrich^®^, Catalog Number 242241) is derived from lignite coal; in this study it was used solely for comparison purposes as its main application is the removal of organic impurities from water. It is a granular material and as pretreatment was sieved to a size range of 0.5–1 mm. Morphological characteristics, porosity and surface area were experimentally determined as reported in the following paragraphs. AC toluene adsorption behavior has been previously investigated^34^ and the optimized parameters for the kinetic and thermodynamic relationship were herein adopted for comparison purposes.

#### Biochar

Biochar was obtained as residue from the V 3.90 Burkhardt wood gasifier, together with the ECO 180 HG combined heat and power plant. ECO 180 HG commercial gasification plant that consists of a V 3.90 wood gasifier, manufactured by the German company Burkhardt GmbH, coupled with a cogeneration unit manufactured by Leroy-Somer LSA 46.2 with a Man D26 motor. Typical technical data of the ECO 180 HG module include electricity production of 180 kWe, heat production of 270 kWt and consumption of wood pellets (pine wood, DIN A1 quality, diameter 6 mm, length 3.1–40 mm, heating value min 16.5 MJ kg^−1^, ash max 0.7%) 110 kg h^−1^ (8% humidity). Wood pellets are subjected to the gasification process in sub-stoichiometric levels of oxygen; part of the pellet mass is burned and produces heat, gasifies the remaining biomass. The gasification and pyrolysis process takes place at temperatures of approximately 850 °C and results in gas with an average composition of 28% CO, 19% H_2_, 2% CH_4_ and 11% CO_2_. The production of BC residue is 2 kg h^−1^, or approximately 15–16 t year^−1^. These units are currently running in Germany, Italy and Austria; the total estimated amount of this BC available is more than 600 t year^−1^ (European scale). A sample of biochar used in this study was obtained from installation of two V 3.90 Burkhardt wood gasification units located in Plößberg bei Tirschenreuth, Germany that are producing next to 360 kW electrical power and 540 kW thermal power also about 32 tons of biochar. The elemental analysis of this biochar shows on average 78% C; 4.18% Ca; 1.48% K; 0.67% Si; 0.64% Mn and 0.46% Fe as the main components. Obtained BC is a powder material and no pretreatments were carried out prior to experimentation and testing. BC was stored in the desiccator to avoid humidity adsorption.

### Batch configuration

#### Kinetic tests

Batch configuration was carried out to compare different material behavior. The kinetic performance of BC was investigated under different conditions: deionized water and synthetic seawater. Sigma Aldrich^®^ Sea Salts was used to simulate seawater composition. Sea Salts is an artificial salt mixture closely resembling the composition of ocean salts; 40 g L^−1^ of Sea Salt was dissolved in deionized water for at least 24 h (magnetically stirring) to allow the complete dissolution of the salts.

Toluene was used as the target hydrophobic organic pollutant to evaluate the adsorption properties of the BC compared with the AC properties.

Monocomponent solution was prepared in a 1 L glass bottle by spiking toluene in deionized water or synthetic seawater, depending on the test salinity, to obtain a concentrated solution at approximately 350 mg L^−1^ (toluene solubility 535 mg L^−1^). The solution was placed in a closed bottle and left magnetically stirring for 24 h to allow complete toluene dissolution. The solution was finally stored in a *tedlar bag* (5 L) to avoid any volatilization in the head space (room temperature 25 °C).

An aliquot of concentrated toluene solution was sampled from the *tedlar* bag by a glass syringe to prevent any toluene adsorption onto the syringe surface and spiked in the glass vials, where the actual tests were carried out. The precise sorbent material amount was placed into the vials; after that, the glass vials were sealed by a Teflon face gray buthyl stopper (Wheaton, Millville, NJ) and crimped by an aluminum cap. This setup was used to avoid any toluene lost during the tests.

Bottles for kinetic and equilibrium tests were mechanically stirred (15 RPM), and the batch tests were performed at room temperature (25 °C). Kinetic tests were carried out to investigate the materials’ kinetic performance and to assess the equilibrium time required for the following isotherm tests. For kinetic tests, 1 g L^−1^ was chosen as a solid/liquid ratio (0.02 L of contaminated solution was placed in contact with 0.02 g of sorbent material).

An aliquot of the solution was sampled at the test beginning (that is, time 0) to evaluate the starting toluene concentration (C_0_), and other sampling was carried out to investigate C_(t)_ after 0.25, 0.5, 1, 2, 3, 4, 5, and 6 h. It must be specified that an additional sampling was carried out after 24 h (C_t=24h_) to confirm the attainment of equilibrium.

#### Equilibrium tests and parameters

The same kinetic setup was chosen to carry out the equilibrium (isotherm) tests: an aliquot of solution was sampled at the test starting time (t = 0) to evaluate the toluene initial concentration C_0_, and the other sampling was carried out after 24 h to ensure the achievement of equilibrium. The results of previous kinetic tests revealed that 24 h can be considered sufficient to reach equilibrium.

The equilibrium sorbed concentration q_e_, expressed in mg g^−1^, was calculated using the following eq. (1):


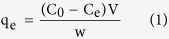


where C_0_ is the starting toluene concentration expressed in mg L^−1^, q_e_ is the toluene sorbed amount in mg g^−1^, C_e_ is the equilibrium toluene concentration expressed in mg L^−1^, V is the volume of the solution in L and w is amount of sorbent in g.

Equilibrium tests were carried out while maintaining constant the sorbent-solution ratio and changing the initial toluene concentration. Isotherms were performed by using 1 g L^−1^ as a solid/liquid ratio (0.02 L in contact with 0.02 g of sorbent material). BC equilibrium performance was investigated for toluene concentrations between 35 and 350 mg L^−1^.

### Analytical methods

#### Gas chromatography (GC)

Toluene was determined by gas chromatography (DANI GC 1000 equipped with a DANI 86.50 headspace auto sampler, Milan, IT) using a capillary column (75 m length, 0.53 mm ID, TRB 624) and a flame ionization detector (FID). An aliquot of 100 μL of the sample was diluted with 3 mL of deionized water and placed in a 10-mL headspace glass vial sealed with a Teflon-faced butyl septum. The gas chromatography conditions were as follows: splitless injection, 180 °C injector temperature, helium carrier gas (flow 14 mL min^−1^), air and H_2_ for the FID (flows 1.1 mL min^−1^ and 0.65 mL min^−1^, respectively), 250 °C detector temperature.

The oven temperature was programmed as follows: 60 °C holding for 0.5 min, increasing by 6 °C per minute to 110 °C holding for 0 min, then increasing by 15 °C per minute to 180 °C holding for 0 min.

The headspace analysis program was performed as follows: manifold temperature 75 °C, transfer line temperature 180 °C, shaking softly.

The GC was previously calibrated with standard toluene concentrations over a linear response range.

#### Scanning Electron Microscope (SEM)

A Zeiss Auriga FESEM has been used. SEM analysis was performed to evaluate the morphology of the materials. The analyses were carried out on the as-received materials (without any pretreatment).

#### Porosimetry

Surface area, the Brunauer–Emmett–Teller (BET) multipoint method^35^ and textural analysis were determined using N_2_ adsorption/desorption measurements at liquid nitrogen temperature (−196 °C), using Micromeritics ASAP 2010 equipment. Samples were pretreated under vacuum at 200 °C for 2 h. The pore distribution was determined by the Barret–Joyner–Halenda (BJH) method^36^ and by the Horvath Kawazoe (HK) equation^37^ from the adsorption isotherm. The analysis of the micropore isotherm was performed by the t-test^38^ taking the curve of Harkins and Jura^39^; the total pore volume was determined by the rule of Gurvitsch^40^.

### Modeling and calculation

#### Kinetic modeling

Toluene adsorption kinetic data were interpreted by a specific interaction mechanism between dissolved toluene and active sorbent surface; the data were fitted according to a mathematical model developed for toluene adsorption by using Micromath^®^ Scientist 1.0 for parameter optimization.

The adsorption kinetic expression mechanism was derived from the *Lagergren* pseudo-first order equation^41^, as reported in eq. (2):


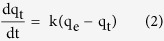


where k is the adsorption kinetic constant, q_e_ is the sorbed equilibrium toluene concentration expressed in mg g^−1^, and q_t_ is the sorbed toluene concentration at time t calculated as follows (3):


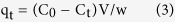


where C_t_ is the sorbed toluene concentration in the water phase at time t. The model was explained using the following boundary condition: negligible external transport due to the high turbulence given by the stirring condition, at t = 0, q_t_ = 0, whereas at t > 0, q_t_ > 0. The kinetic adsorption is therefore linearly dependent on the adsorption driving force (given by the difference between q_e_ and q_t_).

By integrating eq. (2) between q_t = 0_ and q_t_ and between 0 and t, the kinetic equation for toluene adsorption becomes (4)


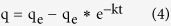


k and q_e_ were optimized via the nonlinear regression of q_t_ vs t experimental data according to model (3).

#### Equilibrium modeling

Equilibrium tests (isotherms) were carried out to investigate BC sorption behavior and thus its affinity for the target hydrophobic contaminant; additionally, isotherms are useful to compare BC performance with more consolidated sorbent materials such as AC.

Two isotherm models were used for equilibrium data fitting purposes: the Langmuir and the Freundlich models. The models were applied to each experimental plot in deionized water and synthetic seawater, respectively, to evaluate which one better simulates the material adsorption behavior. The Langmuir and Freundlich models are reported in eqs (5) and (6), respectively:


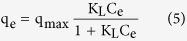



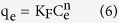


where q_max_ (mg g^−1^) is the maximum adsorbable amount, K_L_ is the Langmuir thermodynamic constant (L mg^−1^), K_F_ is the Freundlich constant expressed in L g^−1^ and n is a dimensionless parameter greater than zero; n > 1 means upwards concavity, whereas n < 1 represents downward concavity. Usually, n is an empirical parameter, although some authors have given it a physical meaning as a measure of the surface heterogeneity^42^.

It must be specified that the thermodynamic modeling (isotherm) was carried out only for comparison purposes. Thus, no specific physical meaning has been attributed to the optimized parameters.

## Results

### SEM analysis

**Figure 1:**
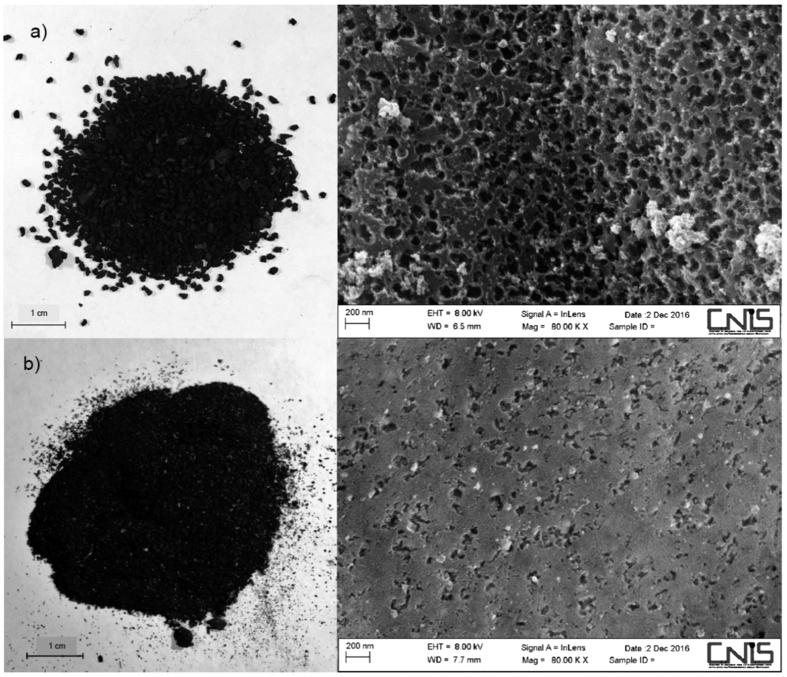
Optical and SEM images of (**a**) AC and (**b**) BC. The authors wish to acknowledge the CNIS for the SEM images.

In order to compare the morphology of the tested sorbent materials, SEM analyses were carried out. AC and BC are shown in Fig. 1a,b respectively. The surface of the AC sample appears highly homogeneous; the pores are regularly disposed onto the surface (40–100 nm on average). On the other hand, the morphology of the BC is more difficult to analyze due to its heterogeneity; the pores distribution is also extremely heterogeneous in term of shape and dimensions, ranging from 25 to 140 nm. The amount of meso- and macropores is much higher for AC than for BC; no informations on the micropores distribution can be obtained with the SEM analyser.

The carbonaceous materials’ texture and development of porosity was strongly affected by the characteristics of the starting materials. The activated carbon (Sigma Aldrich) is a material usually used as target sorbent for lab scale study; it is obtained in controlled conditions, and consequently its texture is rather homogeneous. The BC material is the solid residual of a high temperature gasification process; this involves in a more heterogeneous texture and a greater variety of randomly distributed pore size than the AC material.

#### Textural characterization

**Figure 2:**
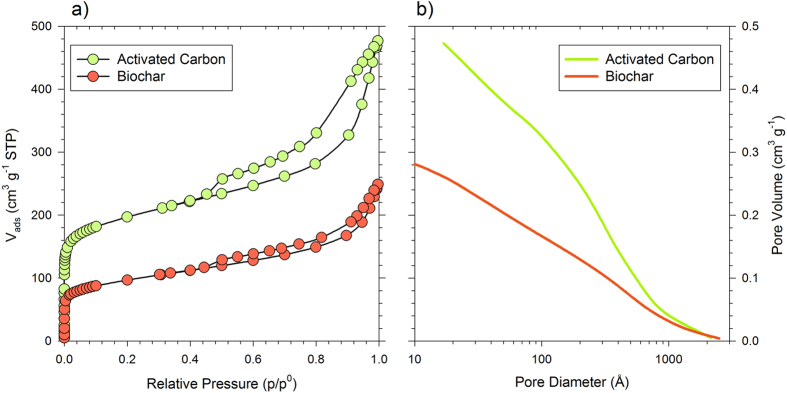
Pore structure. (**a**) N_2_ adsorption–desorption isotherms at −196 °C; (**b**) total pore volumes.

Both the biochar and the activated carbon Norit, type Darco^®^ are micro-mesoporous materials that have similar pore structures, as shown by the N_2_ adsorption/desorption isotherms (type IV, hysteresis loops type H4; see Fig. 2a). The specific surface area and the pore volume values have been esperimentally determined and are reported in Table 1. In both materials, the high surface area values are mostly due to micropores, and the great total volumes (Fig. 2b) are principally related to mesopores, but in the AC Norit, the amount of micro- and mesopores is almost twice that of the BC, confirming the results obtained from the SEM analysis.

Regarding the pore size distribution, for both materials, meso- and macropores are continuously distributed in a 20–1000 Å range and are most abundant in the 20–100 Å range. Using the slit-shaped HK model, typical for graphitic materials, the mean dimensions of micropores, which correspond to the mean distance between carbon planes, are equal to 6.6 Å for the BC and 6.2 Å for the AC Norit. These data indicate a close similarity in the porous structure of the materials, although the AC Norit is more porous than the BC.

#### Kinetic tests

**Figure 3:**
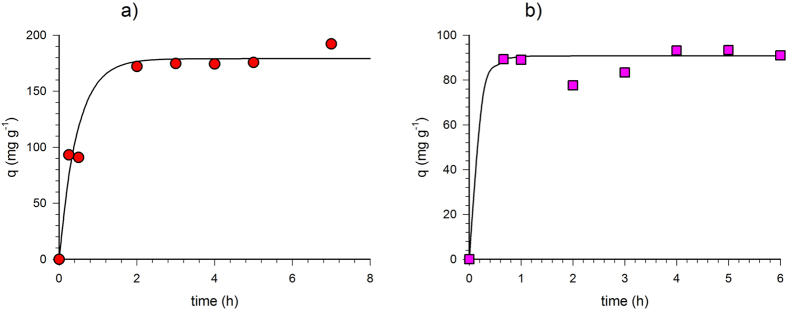
Toluene kinetic tests onto BC. Experimental data vs calculated behavior according to eq. 4 in deionized water (**a**) and synthetic seawater (**b**).

BC adsorption kinetic test data are reported in Fig. 3a,b along with the behavior predicted by the kinetic model (eq. (4)), respectively in deionized and synthetic sea water. Kinetic constants have been optimized by nonlinear regression according to eq. (4). Table 2 reports optimized values of k and q_e_ in comparison with the previously determined ones for AC^34^.

The BC kinetic data in deionized water (Fig. 3a) are clearly well represented by the Lagergren model (eq. (4)) as also confirmed by the high calculated regression coefficient values (R^2^) and the correlation factor (Table 2). Moreover, the figure shows that the toluene removal equilibrium is very quickly reached and after 2 h the sorbed concentration remains constant.

Comparing the kinetic optimized parameters (Table 2) with the previously obtained AC ones^34^, it is clear that the toluene removal on the BC surface is faster than on the AC surface as indicated by the significantly higher k value.

Possible effects of the salinity on kinetic performance have been investigated. BC kinetic results carried out in synthetic seawater are shown in Fig. 3b. In addition, in this case, data are sufficiently represented by the adopted kinetic model. This consideration is also confirmed by the high R^2^ values and by the correlation factor (Table 2).

The kinetic test carried out in synthetic seawater solution confirms the same consideration made for the deionized water tests. The equilibrium is quickly reached and after only 1 h the sorbed concentration remains constant for the length of the test. Moreover, toluene removal on BC is again faster than on the AC surface (as derived from Table 2) and the increase in the kinetic constant, k, appears in the same order as obtained in deionized water.

The salinity has a positive effect on the k value; indeed, a kinetic constant increase has been calculated with respect to the deionized water for both AC and BC with a slightly larger effect on BC than on AC (20% vs 13%, respectively).

The higher removal kinetic by BC with respect to AC can be very useful for environmental application because it entails less time required to reach the fixed objective. In addition, the kinetic and salinity increase is certainly an advantage for real application where high ionic concentrations usually occur.

#### Equilibrium tests

Isotherm equilibrium tests have been carried out to investigate the material adsorption property at equilibrium and their affinity for the selected contaminant.

**Figure 4:**
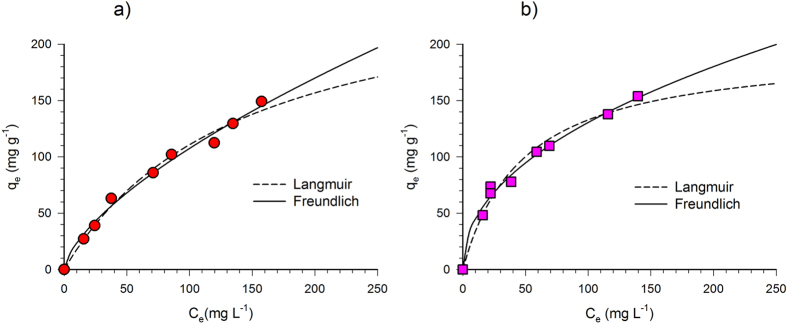
Toluene equilibrium tests onto BC. Experimental data vs calculated behavior according to Langmuir and Freundlich models in deionized water (**a**) and synthetic seawater (**b**).

Toluene adsorption equilibrium tests carried out in deionized water on BC have been modeled with the Langmuir and Freundlich models; the optimized parameters, the regression coefficients R^2^ and the correlation factors for the Langmuir and Freundlich models are reported in Table 3 in comparison with the previously determined values for AC^34^. BC data are represented in Fig. 4a,b (respectively in deionized and synthetic sea water) along with the behavior predicted by the adopted models.

As shown in Fig. 4a, the equilibrium data are well fitted with both Langmuir and Freundlich models in deionized water. This peculiarity is also confirmed by the high R^2^ and the high correlation factor reported in Table 3 for both models. By this regard, the dual behavior has been already reported for activated carbon^34^. On the other hand, it has to be noticed that the investigated concentration range, experimentally limited by the toluene aqueous solubility, is still far from a potential plateau which is a peculiar characteristic of the Langmuir isotherm.

The same behavior is observed for the test carried out in synthetic sea water (Fig. 4b) as both isotherms well represent the experimental data and the adsorption capacity of the BC seems to be still far from a maximum value.

Comparing the BC thermodynamic constants (Table 3) a significant positive effect of salinity is clearly observed. Indeed, K_L_ and K_F_ values both increase around 3-fold from deionized to synthetic sea waters. Consequently, at the lower concentration range the toluene adsorption onto BC is significantly higher in synthetic sea water than in deionized water, whereas at the highest investigate range the adsorption becomes similar in the two conditions.

## Discussion

**Figure 5:**
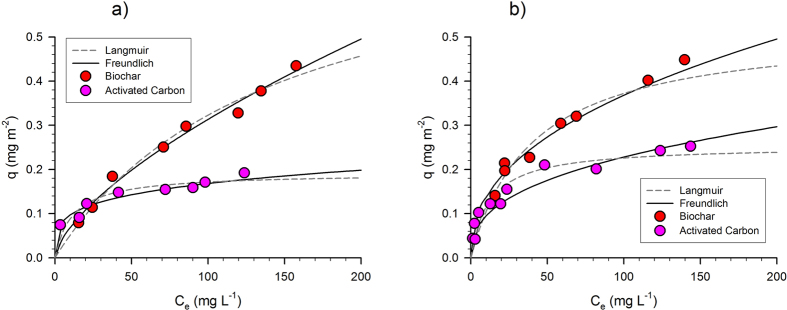
Toluene equilibrium tests onto BC and AC. Experimental data, normalized to the specific surface area, vs calculated behavior according to Langmuir and Freundlich models in deionized water (**a**) and synthetic seawater (**b**).

The sorbent surface area of carbonaceous materials usually plays a main role in the adsorption of dissolved organic compounds^17^,^43^. In order to compare the adsorption performances of the investigated materials, BC and AC experimental and predicted sorption behavior has been normalized for the total measured specific surface area (712 and 343 m^2^g^−1^ for AC and BC, respectively) and reported in Fig. 5a,b (for deionized and synthetic seawater respectively). The figures clearly show that, in both solutions, toluene adsorption onto BC is significantly higher than that onto AC especially at the highest equilibrium concentration level.

On the other hand, the experimental characterization carried out on both materials has clearly shown the similarity in their porous structure. Indeed, even though the surface area and total pore volume of AC is 2-fold than BC, their distribution among micropores and meso-macropores is almost the same.

Because the amount of toluene adsorbed is not directly related to the material surface area or to the distribution of the total porosity among micropores and meso-macropores, these results clearly indicate that more specific contaminant-surface interactions should be considered.

By this regard, Daifullah and Girgis^44^ investigated the benzene, toluene, ethylxylene and xylene (BTEX) adsorption onto carbonaceous materials; they demonstrated that it is a function of the complexity of the carbon surface, such as the high content of oxygen functionalities, beyond the porosity. Moreover, Xu and coworkers^32^ demonstrated that the mercury, Hg (II), adsorption onto hickory chip-derived biochar is controlled by π binding. This theory has been further used to describe the organic compound adsorption onto different sorbent materials with respect to AC; other studies have indeed demonstrated that the occurrence of π interactions between hydrophobic organic compounds and the surface of carbonaceous materials involves the establishment of stronger sorption interactions^45^. Thus, the observed higher BC adsorption capacity with respect to AC may be explained by the occurrence of stronger and more specific interactions between toluene and the BC surface.

The observed faster kinetic and higher sorption capacity of BC with respect to AC are very promising for the prospective use of BC as a sorbent material. The activated carbon is moreover a manufactured sorbent material that has to be produced *ad hoc* for these kind of applications, whose production usually requires a higher temperature and a surface activation procedure^32^, which is connected to a higher cost. The biochar used in this study is instead waste of the wood gasification for the production of electricity, so its cost of production is much lower than that of AC. Furthermore, the use of BC instead of a manufactured material involves a lower contribution to global warming.

In conclusion, the use of biochar could be a green and cost-effective substitute for organic compound adsorption from water solution.

## Additional Information

**How to cite this article**: Silvani, L. *et al*. Characterizing Biochar as Alternative Sorbent for Oil Spill Remediation. *Sci. Rep.* **7**, 43912; doi: 10.1038/srep43912 (2017).

**Publisher's note:** Springer Nature remains neutral with regard to jurisdictional claims in published maps and institutional affiliations.

## Figures and Tables

**Table 1:** Textural characterization.

	AC		BC	
	Surface area m^2^ g^−1^	Pore volume cm^3^ g^−1^	Surface area m^2^ g^−1^	Pore volume cm^3^ g^−1^
total	712 ± 2	0.736	343 ± 2	0.383
micropores	501	0.281	224	0.136
meso-macropores	211	0.455	119	0.247

Total, micropores and meso-macropores surface area and pore volume of AC and BC.

**Table 2:** Kinetic parameters.

	AC^34^	BC
Deionized Water		
q_e_ (mg g^−1^)	209 ± 6.81	178 ± 6.67
k (h^−1^)	0.755 ± 0.0982	1.93 ± 0.353
R^2^	0.994	0.993
correlation	0.987	0.981
Synthetic Seawater		
q_e_ (mg g^−1^)	136 ± 5.42	89.4 ± 3.76
k (h^−1^)	0.856 ± 0.143	2.32 ± 0.466
R^2^	0.991	0.991
correlation	0.977	0.968

Biochar adsorption and desorption kinetic constant, with regression coefficient factor for kinetic tests in deionized water and synthetic seawater.

**Table 3:** Isotherm parameters.

	AC^34^	BC
Deionized Water		
Freundlich		
K_F_ (L g^−1^)	40.2 ± 5.07	5.13 ± 1.04
n	0.237 ± 0.0284	0.661 ± 0.0432
R^2^	0.995	0.997
correlation	0.965	0.992
Langmuir		
q_max_ (mg g^−1^)	136 ± 8.16	268 ± 35.81
K_L_ (L mg^−1^)	8.56 × 10^−2^ ± 2.58 × 10^−2^	7.09 × 10^−3^ ± 1.65 × 10^−3^
R^2^	0.991	0.996
correlation	0.939	0.991
Synthetic Seawater		
Freundlich		
K_F_ (L g^−1^)	39.6 ± 6.41	15.4 ± 1.95
n	0.295 ± 0.0388	0.464 ± 0.0291
R^2^	0.977	0.997
correlation	0.909	0.989
Langmuir		
q_max_ (mg g^−1^)	166 ± 10.4	197 ± 15.4
K_L_ (L mg^−1^)	8.59 × 10^−2^ ± 2.31 × 10^−2^	2.07 × 10^−2^ ± 3.82 × 10^−3^
R^2^	0.977	0.995
correlation	0.910	0.980

Adsorption thermodynamic constant, with regression coefficient factor for kinetic tests in deionized water and synthetic seawater.
